# Understanding the Effect of Race on Medicare Advantage Enrollment

**DOI:** 10.1111/1475-6773.14464

**Published:** 2025-03-13

**Authors:** Adam Atherly, Roger Feldman, Eline van den Broek‐Altenburg, Bryan E. Dowd

**Affiliations:** ^1^ Department of Health Administration College of Health Professions, Virginia Commonwealth University Richmond Virginia USA; ^2^ Division of Health Policy and Management School of Public Health, University of Minnesota Minneapolis Minnesota USA; ^3^ Department of Radiology Larner College of Medicine, University of Vermont Burlington Vermont USA

**Keywords:** health plan choice, Medicare, Medicare advantage, racial differences, regression decomposition

## Abstract

**Objective:**

To understand why Medicare Advantage (MA) has a relatively larger market share among racial minorities than traditional Medicare (TM).

**Study Setting and Design:**

We estimate Probit models for the choice of the MA sector versus TM by Black and Hispanic beneficiaries, as compared with White beneficiaries. We use a non‐linear version of the Oaxaca‐Blinder decomposition to decompose differences in the probability of MA enrollment by race into differences in explanatory variable values versus differences in the coefficients on those variables, which we identify as “preferences” for MA.

**Data Sources and Analytic Sample:**

We combined 2020 Medicare Current Beneficiary Survey (MCBS) data with CMS data on MA plan payment levels aggregated to the county level, star ratings, and measures of market competition.

**Principal Findings:**

In the Black/White beneficiary comparison, 83% of the 17% point difference in the probability of MA enrollment was explained by differences in preferences (*p* < 0.001) while only 17% was explained by differences in attributes (*p* < 0.05). In contrast, in the Hispanic/White beneficiary comparison, 72% of the difference was explained by differences in attributes (*p* < 0.001) and 28% was explained by differences in preferences (*p* < 0.01). Attributes associated with differing rates of MA enrollment by race included both market‐level characteristics (e.g., payment levels) and personal characteristics (age, level of pain, and chronic disease count). Preferences associated with differing rates of MA enrollment included coefficients of sector characteristics such as payment rates and the number of four‐star+ plans available and age.

**Conclusions:**

In this study, we find that the higher MA enrollment rate for Black versus White beneficiaries is largely associated with differences in preferences, while the higher enrollment rate for Hispanic beneficiaries is more associated with differences in attributes. Differences in preferences for MA sector characteristics were significant in explaining higher MA enrollment rates for both groups compared with White beneficiaries, suggesting that changes in payment rates will disproportionately impact racial minorities, particularly for Black beneficiaries. However, the reasons for different preferences for MA among racial groups remain somewhat of a puzzle, particularly given that we control for demographics, health, and market characteristics.

**Summary**

- What is known about this topic
  ○ Medicare Advantage (MA) enrollment has been growing rapidly and now exceeds 50% of the Medicare population.
  ○ Racial and ethnic minorities have notably higher rates of MA enrollment than White beneficiaries.
- What this study adds
  ○ Black and Hispanic beneficiaries both have higher rates of MA enrollment than White beneficiaries, but the reasons differ.
  ○ The higher MA enrollment rate for Black beneficiaries is largely explained by different preferences.
  ○ Differences in attributes, particularly income and education, explain the higher MA enrollment rate for Hispanic beneficiaries.

## 1 Introduction

Medicare beneficiaries have a choice between the traditional Medicare (TM) program and private health insurance plans in a program currently called Medicare Advantage (MA). Although MA has existed for nearly four decades, only in the past decade has it become widely popular, with enrollment now exceeding 50% of beneficiaries [1]. Although enrollment in MA has grown across all racial groups, enrollment among racial minorities is notably higher than among non‐minorities [2, 3]. The reasons for this difference in enrollment rates between minority and non‐minority beneficiaries are unclear and are the topic of this paper.

TM operates under a “Fee‐for‐Service” structure, with administratively determined fees that are typically higher than the public Medicaid program but less than commercial insurance [4]. Virtually all providers and hospitals participate in TM, providing high levels of access for participants [5]. However, TM's coverage design is almost unchanged since the 1960s and includes extensive out‐of‐pocket payments for services and coverage gaps [6]. Private “Medigap” insurance policies are available, for an additional charge, to cover the gaps in TM [7]; alternatively, lower‐income Medicare beneficiaries can become “dually eligible” and enroll in the Medicaid program in addition to Medicare to gain additional coverage.

The other alternative to reduce TM cost sharing is MA. The MA program was introduced in 1985 to give Medicare beneficiaries an alternative to TM with choices that are more similar to those available to workers with employment‐based health insurance [8]. MA offers beneficiaries a choice of available private health plans. Similar to employer‐based plans, there is an annual open enrollment period. Outside the open enrollment period, it is possible to switch to another MA plan or TM under some circumstances. MA plans have a number of advantages and disadvantages compared to TM. MA plans offer expanded benefit packages, lower cost‐sharing for covered services, and lower overall financial risk, while potentially requiring narrower provider networks and more care management than TM [9].

MA enrollees have historically tended to be younger and healthier than TM beneficiaries, based on self‐assessed health, functional status, and cognitive functioning. Enrollment tends to be “sticky,” with many enrollees remaining in the same plan over time [10]. Enrollees are less likely to be below the federal poverty line (FPL), but more likely to be between 100% and 200% of poverty [11]. MA enrollees tend to select plans with lower premiums and higher benefits [12, 13, 14, 15], with lower out‐of‐pocket costs of particular importance [16]. Enrollment also is higher in plans with better star ratings and quality metrics [17, 18]. Disenrollees from MA to TM tend to be higher cost and sicker than beneficiaries continuously enrolled in TM [19, 20, 21, 22, 23, 24], although most beneficiaries who disenroll from an MA plan enroll in a different MA plan rather than TM [17].

MA plans' market areas are based on counties, with plans allowed to select which counties they enter. Plans are more likely to enter counties with higher plan payment rates [25, 26], which leads to both higher levels of plan competition and higher enrollment in high payment counties [27]. The effect of overall payment levels on program participation and benefits offered by MA plans is unclear—payment cuts associated with the Balanced Budget Act of 1997 led to considerable instability in the program, with enrollment declines and plan withdrawals [28, 29], while payment reductions in the Affordable Care Act (ACA) did not. Indeed, MA market share more than quadrupled between 2005 and 2024 [30, 31] and in 2023, for the first time, more than half of beneficiaries were enrolled in MA [1].

Enrollment in MA plans is particularly strong among beneficiaries who belong to racial minorities. In 2023, while 52% of all beneficiaries enrolled in MA plans, 59% of Black Medicare beneficiaries and 67% of Hispanic Medicare beneficiaries joined an MA plan, compared to 43% of White beneficiaries [1]. The reasons for this difference are unclear. The observed differences in MA enrollment by race could be a function of either differences in attributes of the MA sector or differences in preferences for MA. Previous research has found that overall MA market share grew over the past 15 years because of increasing preferences for the program among Medicare beneficiaries, rather than changes in program attributes or beneficiary characteristics [32]. Similarly, minority beneficiaries could have a “taste” for MA—a stronger probability of enrollment controlling for differences in program attributes and beneficiary characteristics—or differences in program offerings and beneficiary characteristics could lead to differences in the probability of enrollment. For example, minority beneficiaries are more likely than White beneficiaries to live in urban areas and to be lower income. Both factors are associated with a higher probability of MA enrollment. Two important elements are lower incomes among minority beneficiaries and higher plan payment rates in areas with higher concentrations of minorities [33]. MA plan enrollment by race generally matches the demographic makeup of plans' areas of operation [34].

Why would the “taste” for MA differ between minority and non‐minority beneficiaries? One way this could occur is if quality or access to care for minority beneficiaries within TM is lower relative to MA. Disparities in access to and outcomes of care in TM are well documented [2, 35, 36, 37]. But the impact of enrollment in MA plans on racial disparities in access and outcomes is less clear. MA plans have reduced or eliminated disparities in access to care for minorities for some services, particularly for mammography, preventable hospitalizations, and hospital readmissions [38]. There is also evidence that Black beneficiaries in MA plans may have a longer life expectancy than do Black beneficiaries in TM [39]. A recent review by the Kaiser Family Foundation identified 62 studies that compared MA plans to TM on disparities in access and outcomes of care by race, although only 12 were published after 2016 [40, 41]. Overall, the review found Black enrollees in MA plans had lower disparities in having a usual source of care, care transitions, and access to prescription drugs but no difference in wait times, finding a new provider, and overall satisfaction with care coordination.

Much of the work to date has focused on Black/White beneficiary differences, with less focus on Hispanic/White beneficiary differences, although Hispanic beneficiaries have the highest MA enrollment rate of the three groups and are the largest racial minority group in the United States. Indeed, recent work suggests that MA plans may eliminate some disparities between Hispanic and White beneficiaries (delaying care due to cost and not reporting problems paying medical bills), but not Black and White beneficiaries [2].

But the reasons for higher enrollment among racial minorities in MA plans remain unknown and are the focus of this paper.

## 2 Methods

### 2.1 Data Sources

Our main data source is the 2020 Medicare Current Beneficiary Survey (MCBS), a representative rolling cohort survey of Medicare beneficiaries. CMS conducts the MCBS annually and uses it in operational guidance for the Medicare program. It is the most comprehensive source of data on Medicare spending [42]. MCBS has an overall response rate of 60% for the initial survey and a response rate of over 80% for subsequent rounds; prior research has found that procedures employed by the survey reduce or eliminate bias in the survey due to non‐response [43].

Our sample includes individuals enrolled in Medicare Parts A and B for all months of Medicare eligibility. The total sample size was 15,476. Following the literature on Medicare health plan choice, we excluded individuals under age 65 (disabled) (*n* = 2577), currently employed individuals, and ESRD‐entitled beneficiaries. For our Probit model (described below), given an effect size of 17% points (for the Black–White comparison) and a *p* value of 0.05, the statistical power of the model is above 90%.

We supplemented the MCBS with 2020 CMS data on county‐level MA payment rates, enrollment by plan/contract, and Part D premiums. The CMS data were combined with the MCBS at the county level, while the Part D premium data were combined at the regional level (CMS establishes 34 Part D regions). Both the MA and Part D averages were weighted by plan enrollment. The unit of analysis is the individual, with the supplemental variables based on the county of residence.

### 2.2 Statistical Model

Our approach is based on a Blinder‐Oaxaca regression decomposition (hereafter referred to as BO [44, 45]), which partitions changes in an outcome of interest between two groups into differences in explanatory variables versus differences in the coefficients of those variables. The BO methodology has been used extensively to study gender differences in labor market outcomes [46, 47], health disparities [48], health care spending [49], and obesity [50, 51]. BO decompositions assume that choices are a function of “attributes”—that is, the values of the variables—and “preferences” or the coefficients of the variables.

The model estimates the impact of individual and market attributes on the probability of picking the MA sector. We decompose the difference in the probability of choosing the MA sector between different racial groups. In a linear model, using Black–White as the example, that difference can be written as:

(1) $${\hat{y}}_{\text{White}}-{\hat{y}}_{\text{Black}}=\bar{x}\prime_{\text{White}}\beta_{\text{White}}-\bar{x}\prime_{\text{Black}}\beta_{\text{Black}}$$

Equation (1) shows the difference in MA market share between Blacks and Whites evaluated at the mean values of the explanatory variables ($\bar{x}$) and the coefficients of the explanatory variables (β). Equation (1) can be rearranged as:

(2) $${\hat{y}}_{\text{White}}-{\hat{y}}_{\text{Black}}=\left(\bar{x}\prime_{\text{White}}-\bar{x}\prime_{\text{Black}}\right)\beta_{\text{White}}+\bar{x}\prime_{\text{Black}}\left(\beta_{\text{White}}-\beta_{\text{Black}}\right)$$

Equation (2) decomposes the MA market share into two parts. The first part represents the difference in market share due to differences in the explanatory variables, evaluated at the “White” coefficients. The second part shows the difference in market share due to differences in the coefficients, evaluated at the “Black” values of the explanatory variables.

Our statistical model is a univariate Probit model of the individual's choice between the MA sector and TM, controlling for the individual and market factors listed below. Probit is a standard approach to estimating models with binary dependent variables. It models the probability that the outcome equals 1 and assumes a standard normal distribution as the link function. Formally,

(3) $$P\left(y=1|X\right)=\Phi \left(\beta_{0}+\beta_{1}x_{1}+\beta_{2}x_{2}+\ldots +\;\beta_{\kappa }x_{k}\right)$$

where *P*(*y* = 1|*X*) is the probability the outcome is equal to 1, *X* is the explanatory variables and Φ() is the CDF of the standard normal distribution. We calculated marginal effects for each coefficient. We estimated the model using Stata version 18.

### 2.3 Variables

We used individual and market characteristics suggested by the prior literature as important predictors of MA choice, with hypotheses about the direction of the estimated coefficients based on the prior literature [8, 10, 11, 12, 13, 14, 15, 16, 17]. Variables include demographic characteristics at the individual level: age (continuous, in years), annual income from all sources as a percentage of the Federal poverty level (FPL), education (dichotomized as high school graduate (yes/no), college graduate yes/no with did not graduate from high school as the reference category) and indicator variables for female, married, and living in an urban area. Prior research has found that age and education have a negative relationship to the probability of MA enrollment; female, married, and urban area have a positive relationship. Individuals from 100% to 250% of the FPL have a higher probability of enrollment than do those above or below that income range.

Given the extensive literature on the effect of health on MA enrollment, we included measures of self‐rated health (on a 1–5 scale from excellent to poor) and a count of chronic illnesses from a list of approximately 20 possibilities, including asthma, arthritis, heart disease, and cancer. We hypothesized that health would be negatively correlated with the probability of enrollment.

We also included measures of MA market structure: the county MA benchmark payment, the number of MA contract options in the individual's county of residence, and the average Part D premium. All of these variables have been found to have a positive relationship with the probability of MA enrollment in prior research [12, 14, 15, 16, 17, 18]. The county MA benchmark is correlated with plan benefits [18], while having more plans to choose from within a county increases the likelihood of a match between beneficiary preferences and plan options. We did not include the count of zero‐premium plans or the average premium because the former is highly correlated with the total plan count and the latter lacks variation because most plans are zero‐premium [1]. The Part D premium represents the cost of obtaining prescription drug coverage in TM. It is expected to have a positive coefficient, assuming TM and MA are economic substitutes.

To control for the availability of other coverage options, we included an indicator for employer supplemental coverage (which can take many forms) and eligibility for Medicaid based on income and state of residence. We included a count of the number of MA plans in the county with a quality rating of 4.5 stars or higher. This indicates the quality of MA plan options for the beneficiary, plus the highly rated plans receive a bonus payment that may lead to improved benefits and therefore a higher probability of enrollment [19]. Finally, we included a count of the number of Special Needs Plans (SNPs) in the county of residence for similar reasons.

We also included three other variables from the MCBS that have not (to our knowledge) been used in prior studies of Medicare plan or sector choice. First, an indicator for the presence of a computer in the home measures the beneficiary's ability to research, select, and change plans. Much of the information on differences in plans is available on the internet, so theoretically easier access to the internet should facilitate easier plan comparisons. Ease of comparing and selecting MA plans should lead to a higher probability that the beneficiary chooses the MA sector, which is our choice of interest.

Second, a measure of the presence of chronic pain on a 1–5 scale represents another aspect of health that could affect insurance coverage decisions. Finally, we included an indicator for an inability to drive an automobile. Transportation has been identified as an important element of health care access and is sometimes paid for by MA plans, but not TM, suggesting that individuals with transportation limitations may prefer MA plans. All of these variables could vary by race and may impact choices.

Race was categorized using the Research Triangle Institute (RTI) race codes of White, Black, and Hispanic. The RTI race code is based on an algorithm that creates a more precise measurement of beneficiaries who identify as Hispanic or Asian [52]. It has better validity (versus self‐reported race) than the alternative race measures available in MCBS [53]. It has historically been used by the Social Security Administration, and therefore it is in the CMS enrollment database. There are other racial categories in the data, including Other, Unknown, and Asian, but the MCBS lacks a sufficient sample size to estimate these models.

## 3 Results

The overall MA market share in the unweighted sample was 41% and varied by racial category (Table 1). Overall, 38% of White beneficiaries in the sample were enrolled in MA plans, compared to 55% of Black enrollees and 58% of Hispanic enrollees; both differences were statistically significant (*p* < 0.001). Most individual characteristics varied significantly across racial categories, with White beneficiaries being the oldest category, least likely to be female, most educated, and least likely to live in an urban area. Black beneficiaries were the most likely to be female and had the youngest average age, while Hispanic beneficiaries had the lowest level of education. White beneficiaries were the least likely to be Medicaid eligible and had the lowest rates of poverty (below 100% of FPL) and “near poor” (100%–199% of FPL), while Hispanic beneficiaries had the highest rates of poverty and Medicaid eligibility, and Black beneficiaries had the highest rates of being near poor. Black beneficiaries also exhibited the worst health measured by both the count of chronic diseases (2.7) and by self‐rated health (2.8/5), while White beneficiaries had the best health by both measures. Black beneficiaries were the most likely to be unable to drive (23%), while Hispanic beneficiaries were least likely to have a computer in the home (54%). Hispanic beneficiaries lived in counties with the most plan options (an average of 60.5), most 4.5‐plus star plans (11.0), most SNPs (16.6), and the highest average county MA benchmark payment rate ($940 per month).

**TABLE 1:** Descriptive statistics, by race.

	White	Black	Hispanic	Total	*p*
MA enrollee	0.38 (0.48)	0.55 (0.50)	0.58 (0.49)	0.41 (0.49)	< 0.001
Age	78.02 (8.02)	77.12 (8.28)	77.14 (8.34)	77.85 (8.08)	< 0.001
Female	0.56 (0.50)	0.61 (0.49)	0.57 (0.49)	0.57 (0.50)	0.01
Medicaid eligible	0.14 (0.35)	0.42 (0.49)	0.54 (0.50)	0.21 (0.41)	< 0.001
Income below 100% of FPL	830 (7.8%)	327 (29.2%)	537 (40.7%)	1694 (13.0%)	< 0.001
Income 100%–199% of FPL	2477 (23.3%)	382 (34.1%)	389 (29.5%)	3248 (24.5%)	
Income 200%–299% of FPL	1970 (18.5%)	144 (12.9%)	135 (10.2%)	2249 (17.2%)	
Income 300%–399% of FPL	1225 (11.5%)	86 (7.7%)	78 (5.9%)	1389 (10.6%)	
Income above 400% of FPL	4118 (38.8%)	180 (16.1%)	180 (13.6%)	4478 (34.3%)	
High school graduate	0.48 (0.50)	0.47 (0.50)	0.31 (0.46)	0.46 (0.50)	< 0.001
College graduate	0.40 (0.49)	0.24 (0.43)	0.18 (0.38)	0.37 (0.48)	< 0.001
Married	0.52 (0.50)	0.34 (0.47)	0.46 (0.50)	0.50 (0.50)	< 0.001
Employer subsidy	0.02 (0.13)	0.03 (0.17)	0.02 (0.14)	0.02 (0.14)	0.012
Urban area	0.76 (0.43)	0.86 (0.35)	0.92 (0.28)	0.78 (0.41)	< 0.001
Self‐rated general health	2.38 (1.00)	2.84 (1.01)	2.81 (1.10)	2.46 (1.03)	< 0.001
Chronic pain	0.24 (0.43)	0.23 (0.42)	0.23 (0.42)	0.24 (0.43)	0.234
Count of chronic diseases	2.45 (1.77)	2.71 (1.65)	2.46 (1.75)	2.47 (1.76)	< 0.001
Not able to drive	0.11 (0.32)	0.23 (0.42)	0.18 (0.38)	0.13 (0.34)	< 0.001
Computer in home	0.60 (0.49)	0.35 (0.48)	0.27 (0.44)	0.54 (0.50)	< 0.001
Count of MA plans in county	38.60 (21.36)	40.15 (23.81)	60.46 (29.45)	40.94 (23.45)	< 0.001
Count of 4.5 plus star plans in county	6.64 (5.78)	6.32 (5.59)	11.03 (8.04)	7.06 (6.17)	< 0.001
County MA benchmark	934.35 (30.34)	934.03 (33.10)	940.28 (28.92)	934.81 (30.51)	< 0.001
Monthly average part D drug premium	30.92 (2.91)	31.00 (2.84)	31.54 (3.02)	30.99 (2.92)	< 0.001
Count of SNP plans in county	8.02 (7.39)	10.02 (8.59)	16.66 (12.43)	9.06 (8.55)	< 0.001
*N*	10,620 (81.3%)	1119 (8.6%)	1319 (10.1%)	13,058 (100.0%)	

*Note*: Mean value/count with standard errors in parentheses.

Table 2 shows a Probit regression for MA/FFS plan choice, as a function of race and other characteristics, with the coefficients reported as marginal changes in the probability of MA enrollment. The coefficient on Black is positive (0.118) and statistically significant (*p* < 0.001), indicating that Black beneficiaries are 11.8% points more likely to join a MA plan than White beneficiaries, controlling for other characteristics. Hispanic beneficiaries are 4.6% points more likely to join MA plans than White beneficiaries (*p* = 0.01), controlling for other factors.

**TABLE 2:** Probit model for the probability of Medicare Advantage enrollment versus traditional Medicare, controlling for race and other factors.

Variable	Average marginal effect	Standard error	*z*‐stat	*p*
Black	0.1181	0.0168	7.05	< 0.001
Hispanic	0.0457	0.0183	2.50	0.0120
Medicaid eligible	0.0609	0.0184	3.30	0.001
Income 100%–199% of FPL	0.0532	0.0215	2.48	0.013
Income 200%–299% of FPL	−0.0137	0.0257	−0.53	0.593
Income 300%–399% of FPL	−0.0694	0.0273	−2.54	0.011
Income above 400% of FPL	−0.1166	0.0254	−4.59	< 0.001
Age	0.0013	0.0006	2.01	0.044
Female	−0.0023	0.0095	−0.24	0.809
High school graduate	−0.0457	0.0155	−2.95	0.003
College graduate	−0.0639	0.0174	−3.68	< 0.001
Married	0.0278	0.0101	2.74	0.006
Employer subsidy	−0.2434	0.0376	−6.48	< 0.001
Urban area	0.0523	0.0133	3.92	< 0.001
Self‐rated general health	−0.0099	0.0051	−1.96	0.050
Chronic pain	0.0133	0.0105	1.27	0.203
Count of chronic diseases	−0.0039	0.0029	−1.36	0.175
Not able to drive	0.0026	0.0141	0.19	0.851
Computer in home	0.0017	0.0107	0.16	0.871
Count of MA plans in county	0.0040	0.0004	10.24	< 0.001
Count of 4.5 plus star plans in county	0.0050	0.0012	4.33	< 0.001
County MA benchmark	−0.0013	0.0002	−8.70	< 0.001
Monthly average part D drug premium	−0.0112	0.0018	−6.29	< 0.001
Count of SNP plans in county	−0.0006	0.0010	−0.63	0.528

*Note*: Chi‐square (24) = 1081.80, Prob > chi‐square < 0.001. *n* = 10,704.

Other factors in Table 2 follow the patterns suggested by previous research. The highest probability of enrollment is for persons between 100% and 199% of FPL—the “near poor” (*p* = 0.013). The probability of MA enrollment declines with income, with individuals between 300% and 400% of FPL having an 8.5% point lower probability of enrollment than those below poverty (*p* = 0.01) and beneficiaries above 400% of FPL having a 13.3% point lower probability of enrollment (*p* < 0.01). MA enrollment also declined with education, with high school graduates having a 4.6% point lower probability of enrollment than the reference group (less than high school education) (*p* < 0.01) and college graduates a 6.4% point lower probability (*p* < 0.01). Both being married (*p* < 0.01) and living in an urban area (*p* < 0.01) were significantly associated with a higher probability of MA enrollment. Having more plans in the county was highly associated with the probability of MA enrollment (*p* < 0.01), as was having more plans with 4.5 or higher star ratings (*p* < 0.01).

Table 3 shows three Probit models with MA enrollment stratified by race. For both Black (*p* = 0.015) and Hispanic (*p* = 0.025) beneficiaries, lower income was associated with a higher probability of enrollment relative to the reference group (below 100% of FPL), but that was not true for White beneficiaries. However, White beneficiaries had very significant negative coefficients for the higher income groups (*p* = 0.013 for Income 300%–399% of FPL and *p* < 0.001 for Income Above 400% of FPL), unlike Black and Hispanic beneficiaries. Similarly, higher levels of education were significantly associated with a lower probability of enrollment for White beneficiaries only (*p* < 0.01 for both High School and College Graduate). The health variables were significant only for White beneficiaries, with both self‐rated health (*p* = 0.03) and the chronic disease count (*p* = 0.04) indicating a negative association between health and MA enrollment.

**TABLE 3:** Probit models for the probability of Medicare Advantage enrollment versus traditional Medicare, stratified by race.

Variable	White		Black		Hispanic	
	Average marginal effect	Standard error	Average marginal effect	Standard error	Average marginal effect	Standard error
Medicaid eligible	0.0600***	0.0215	0.0053	0.0510	0.0521	0.0509
Income 100%–199% of FPL	0.0261	0.0275	0.1210***	0.0499	0.1052**	0.0468
Income 200%–299% of FPL	−0.0198	0.0313	−0.0521	0.0725	−0.1073	0.0727
Income 300%–399% of FPL	−0.0813***	0.0327	−0.0495	0.0842	−0.0568	0.0830
Income above 400% of FPL	−0.1276***	0.0308	−0.1456*	0.0748	−0.1012	0.0716
Age	0.0006	0.0007	0.0049**	0.0021	0.0040*	0.0021
Female	−0.0088	0.0104	0.0535	0.0341	−0.0036	0.0329
High school graduate	−0.0643***	0.0190	−0.0368	0.0414	−0.0004	0.0383
College graduate	−0.0861***	0.0206	−0.0703	0.0523	0.0061	0.0507
Married	0.0216**	0.0111	0.1076***	0.0366	−0.0012	0.0338
Employer subsidy	−0.2795***	0.0457	−0.2152**	0.0942	−0.0445	0.1017
Urban area	0.0594***	0.0143	−0.0455	0.0490	0.1328**	0.0652
Self‐rated general health	−0.0123**	0.0057	0.0156	0.0165	−0.0174	0.0153
Chronic pain	0.0052	0.0114	0.0289	0.0364	0.0726**	0.0364
Count of chronic diseases	−0.0063**	0.0031	0.0014	0.0100	0.0110	0.0092
Not able to drive	0.0107	0.0163	0.0323	0.0396	−0.0600	0.0406
Computer in home	−0.0024	0.0117	0.0465	0.0366	0.0179	0.0391
Count of MA plans in county	0.0043***	0.0004	0.0025*	0.0014	0.0017	0.0012
Count of 4.5 plus star plans in county	0.0051***	0.0013	0.0121**	0.0046	0.0090**	0.0042
County MA benchmark	−0.0013***	0.0002	−0.0008*	0.0005	−0.0017	0.0005
Monthly average part D drug premium	−0.0113***	0.0020	−0.0141**	0.0059	−0.0033	0.0063
SNP plans in county	−0.0033***	0.0011	0.0027	0.0030	0.0084***	0.0024
Sample size	*N* = 8895 LR chi^2^ (22) = 709.50 Prob > chi^2^ < 0.001		*N* = 916 LR chi^2^ (22) = 100.00 Prob > chi^2^ < 0.001		*N* = 893 LR chi^2^ (22) = 192.94 Prob > chi^2^ < 0.001	

* *p* < 0.05.

** *p* < 0.01.

*** *p* < 0.001.

Table 4 shows the Blinder‐Oaxaca decompositions and tests for statistically significant differences in both the coefficients and the attributes. For the Black–White beneficiary comparison, 82.9% of the overall difference in MA market share (0.142% points) is due to differences in preferences (coefficients), while only 17.1% of the difference (0.029% points) is due to differences in attributes. The most important preference is the coefficient on age, which alone accounts for nearly half of the difference in preferences. The coefficient indicating “near poor” income is also larger for Black beneficiaries than for White beneficiaries, as are the coefficients for Married and Female. Among the attributes, the largest differences are for income (with Black beneficiaries more likely to be near‐poor than White beneficiaries) and the county payment rate, which is higher on average for Black beneficiaries than for White beneficiaries.

**TABLE 4:** Oaxaca‐blinder decompositions.

	Black–White		Hispanic–White	
	Characteristics	Coefficients	Characteristics	Coefficients
Overall percentage	17.1	82.9	71.5	28.5
Overall change	0.02920*	0.14162***	0.1261***	0.0503**
Income 100%–199% of FPL	0.0140***	0.0218*	0.0087**	0.0220
Income 200%–299% of FPL	0.0025	−0.0059	0.0085	−0.0191
Income 300%–399% of FPL	0.0021	0.0039	0.0029	0.0025
Income above 400% of FPL	0.0344**	−0.0052	0.0240	0.0094
Medicaid eligible	0.0015	−0.0064	0.0200	−0.0004
Age	−0.0035***	0.3327**	−0.0038*	0.3081
Female	0.0034	0.0346*	−0.0001	0.0029
High school graduate	0.0005	0.0139	0.0001	0.0340
College graduate	0.0119	0.0072	−0.0015	0.0431
Married	−0.0238***	0.0480**	0.0001	−0.0138
Employer subsidy	−0.0029**	0.0013	−0.0002	0.0047*
Urban area	−0.0040	−0.0804**	0.0193**	0.0696
Self‐rated general health	0.0074	0.0674	−0.0076	−0.0172
Chronic pain	−0.0003	0.0064	−0.0019**	0.0216*
Count of chronic diseases	0.0004	0.0193	−0.0002	0.0486*
Not able to drive	0.0041	0.0027	−0.0041	−0.0104
Computer in home	−0.0130	0.0323	−0.0066	0.0155
County MA benchmark	0.00005*	0.4112	−0.0115***	−0.6343
Count of MA plans in county	−0.0008*	−0.0725	0.0268	−0.1002*
Count of 4.5 plus star plans in county	−0.0076**	0.0445	0.0159**	0.0326
Monthly average part D drug premium	−0.0014**	−0.0822	−0.0018	0.2576
SNP plans in county	0.0041	0.0463*	0.0392***	0.1023***
Sample size	11,739		11,939	

*Note*: Coefficients represent the percentage of the difference in enrollment probabilities associated with the characteristics and coefficients.

* *p* < 0.05.

** *p* < 0.01.

*** *p* < 0.001.

In contrast to the Black–White beneficiary decomposition, nearly three‐quarters of the difference in enrollment probabilities between Hispanic and White beneficiaries is due to differences in attributes (71.5%, 0.126% points) rather than differences in preferences (28.5%, 0.05% points). The most important attributes are the counts of SNPs and 4.5‐plus star plans, residence in an urban area, and income. The most important preferences are those for the counts of chronic diseases and SNPs.

## 4 Discussion

Higher enrollment by racial minorities in the Medicare Advantage program has been observed for many years, yet the reasons for this difference have not been well understood. Many hypotheses have been offered, including differences in income, education, urbanicity, or plan payment rates. In this study, we disentangled the potential sources of differences in MA market share among racial minorities compared with White beneficiaries. We found that the reasons for Black–White and Hispanic–White beneficiary differences in enrollment rates are different.

Black–White beneficiary differences in MA enrollment rates are associated with differences in preferences. Of particular note, the estimated coefficients describing the relationship between age and enrollment are markedly different for Black beneficiaries compared to White beneficiaries. There are also important differences in the relationship between income and enrollment, near‐poor Black beneficiaries being more likely to enroll in MA plans than near‐poor White beneficiaries.

In contrast, most of the difference in Hispanic–White beneficiary enrollment is explained by differences in characteristics of beneficiaries, including market areas. SNPs were particularly important in accounting for the Hispanic–White beneficiary differences in enrollment. In many ways, Hispanic beneficiaries select plans similarly to White beneficiaries, with lower incomes, education levels, and factors associated with county of residence largely explaining the differences in enrollment rates. This is not the case for the Black–White beneficiary enrollment difference, which is explained by differences in preferences rather than beneficiary attributes.

Put differently, Black Medicare beneficiaries were 17% points more likely to enroll in MA than White Medicare beneficiaries, while Hispanic Medicare beneficiaries were 24% points more likely [1]. If the observed characteristics in our model were equalized among racial groups, Hispanic beneficiaries would only be 6% points more likely than White beneficiaries to enroll in an MA plan, while Black beneficiaries would still be more than 14% points more likely.

The characteristics explain most of the difference between Hispanic and White beneficiary MA enrollment but not the difference between Black and White beneficiary MA enrollment. The difference in the relative enrollment rates may reflect omitted variables that influence Black beneficiary enrollment versus White beneficiary enrollment but do not influence Hispanic beneficiary enrollment versus White beneficiary enrollment (or influence it much less). Alternatively, the preferences for MA plans may vary by race.

One set of omitted variables that could explain the higher enrollment rate for Black beneficiaries is community‐level factors in Black beneficiary majority areas. These could include an easier ability to acquire knowledge about plans from family, friends, and neighbors (due to higher MA enrollment levels within the community), targeted advertising in Black communities, or a more widespread acceptance of MA plans by providers in Black communities. There are also well‐documented differences in trust in the healthcare system between Black and White beneficiaries that could lead to different insurance‐buying behavior. For example, vaccination rates for influenza [54] and Covid‐19 [55] differ between Black and White beneficiaries.

Another possibility is that unobserved benefits in MA are of particular value to Black beneficiaries. Examples are translation services, help in dealing with paperwork burden, transportation, or membership in fitness clubs.

A final possibility for omitted variables is unobserved barriers to care within TM. Although the MA sector is often viewed as having higher barriers to care, the reviewed literature suggests that Black beneficiaries are more likely to have a usual source of care in MA versus TM [42]. We found that the BO coefficient on age is positive, indicating that the probability of joining a MA plan for an older person is higher for Black beneficiaries than for White beneficiaries. This could be because MA plans have unobserved characteristics that appeal to older Black beneficiaries more than older White beneficiaries. Barriers to care are particularly salient as individuals age, so it is possible that the “preference” for MA among older Black beneficiaries reflects unobserved differences in access to care for older Black individuals. Further research into the reasons for the differences in preferences between Black beneficiaries and other beneficiaries would be valuable to understand whether the differences in preferences reflect unobserved differences in access to care for older Black beneficiaries.

There are a number of limitations to our model. First, we include county‐level MA payment rates as a proxy for the generosity of plans. If important omitted plan variables, like network size, are correlated with race, this could create a bias in the estimated coefficients. We also did not control for eligibility for Veterans Administration or other benefits due to military service. Our sample sizes for both Blacks and Hispanics are much smaller than the sample for Whites, which may limit the precision of some of the estimates. It is also important to note that we model choices using a cross‐sectional analysis. There is a high degree of “stickiness” in MA plan choices, so the observed choices may reflect decisions made in prior years. The coefficients of observed attributes that vary over time, such as age, will reflect both cohort effects and switching behavior. However, the switching rate between FFS and MA is relatively high and is far higher among Black and Hispanic beneficiaries than White beneficiaries. A recent study found that the overall switching rate from FFS to MA in 2021 was 7.8%, while the switching rate from MA to FFS was 1.2% [8]. Switching rates were highest among Black beneficiaries, where (in 2022) 15.6% of eligible beneficiaries switched from FFS to MA and 1.4% of Black beneficiaries went the other direction.

## Conflicts of Interest

The authors declare no conflicts of interest.
